# Effects of partner proteins on BCA2 RING ligase activity

**DOI:** 10.1186/1471-2407-12-63

**Published:** 2012-02-08

**Authors:** Stephanie Bacopulos, Yutaka Amemiya, Wenyi Yang, Judit Zubovits, Angelika Burger, Martin Yaffe, Arun K Seth

**Affiliations:** 1 Sunnybrook Research Institute, 2075 Bayview Avenue, S-238, Toronto, ON M4N 3M5, Canada; 2 Department of Laboratory Medicine and Pathobiology, University of Toronto, Toronto, ON, Canada; 3 Department of Anatomic Pathology, Sunnybrook Health Sciences Centre, Toronto, ON, Canada; 4 Barbara Ann Karmanos Cancer Institute and Department of Pharmacology, Wayne State University School of Medicine, Detroit, MI, USA

## Abstract

**Background:**

BCA2 is an E3 ligase linked with hormone responsive breast cancers. We have demonstrated previously that the RING E3 ligase BCA2 has autoubiquitination activity and is a very unstable protein. Previously, only Rab7, tetherin, ubiquitin and UBC9 were known to directly interact with BCA2.

**Methods:**

Here, additional BCA2 binding proteins were found using yeast two-hybrid and bacterial-II-hybrid screening techniques with Human breast and HeLa cDNA libraries. Co-expression of these proteins was analyzed through IHC of TMAs. Investigation of the molecular interactions and effects were examined through a series of in vivo and in vitro assays.

**Results:**

Ten unique BCA2 interacting proteins were identified, two of which were hHR23a and 14-3-3sigma. Both hHR23a and 14-3-3sigma are co-expressed with BCA2 in breast cancer cell lines and patient breast tumors (n = 105). hHR23a and BCA2 expression was significantly correlated (P = < 0.0001 and P = 0.0113) in both nucleus and cytoplasm. BCA2 expression showed a statistically significant correlation with tumor grade. High cytoplasmic hHR23a trended towards negative nodal status. Binding to BCA2 by hHR23a and 14-3-3sigma was confirmed in vitro using tagged partner proteins and BCA2. hHR23a and 14-3-3sigma effect the autoubiquitination and auto-degradation activity of BCA2. Ubiquitination of hHR23a-bound BCA2 was found to be dramatically lower than that of free BCA2, suggesting that hHR23a promotes the stabilization of BCA2 by inactivating its autoubiquitination activity, without degradation of hHR23a. On the other hand, phosphorylated BCA2 protein is stabilized by interaction with 14-3-3sigma both with and without proteasome inhibitor MG-132 suggesting that BCA2 is regulated by multiple degradation pathways.

**Conclusions:**

The interaction between BCA2 and hHR23a in breast cancer cells stabilizes BCA2. High expression of BCA2 is correlated with grade in breast cancer, suggesting regulation of this E3 ligase is important to cancer progression.

## Background

Breast Cancer Associated gene 2 (BCA2) was first identified in an effort to investigate drivers of breast cancer via the subtractive hybridization [1]. These studies aimed to identify differentially expressed genes between Hs578Bst and Hs578T mammary epithelial cell lines derived from adjacent normal and cancerous tissues respectively [1]. These analyses revealed 950 cDNAs enriched in breast cancer cells [1]. Twenty-eight of the cDNAs were novel genes, including BCA2, a 304 amino acid protein encoding a RING H2-domain [2]. BCA2 is located in a chromosomal region known to be up-regulated in breast cancers as well as a region of genomic instability enriched in cancer driver genes [3]. A number of RING E3 ligases have both oncogenic and tumor suppressing roles in cancer processes, notably MDM2, responsible for regulation of p53 [4]; BRCA1/BARD1, involved in DNA repair [5]; and cCbl, which is responsible for the internalization and degradation of EGFR [6].

**Figure 1:**
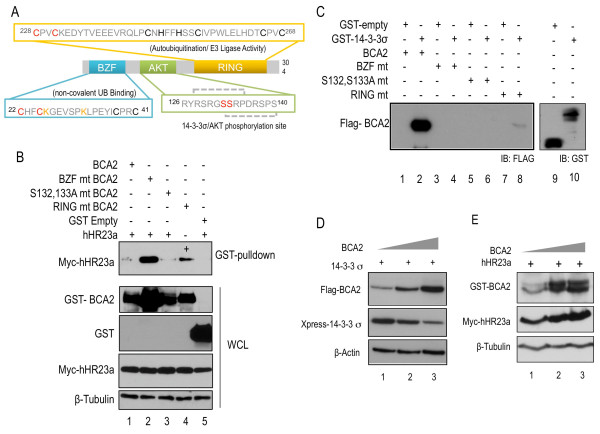
**BCA2 is co-expressed with and binds to both hHR23a and 14-3-3σ**. [**A**] The bolded black amino acids represent key residues which are imperative to the structural integrity of the BZF and RING domains. The bolded yellow residues indicate amino acids which have been modified to alanine by site-directed mutagenesis. The BZF domain (blue) binds ubiquitin and is also the site of BCA2 auto-ubiquitination (ubiquitinated lysine residues are shown in red). The AKT domain (green) is site of AKT-mediated phosphorylation of BCA2, predicted by *in silico *analysis. Serine residues (yellow) are likely phosphorylated, and have been mutated to alanine in the BCA2 S132, 133A mutant. The RING domain (orange) is BCA2 autoubiquitination. The yellow cysteine residues were mutated to alanine to create ligase-dead BCA2 RING mutant. [**B**] Immunoblots of lysates from HEK293T cells which were co-transfected with GST-tagged BCA2, BZF (C22/25A), S132/133A, RING (C228/231A) or GST-empty vector along with myc/his-tagged hHR23A. The top panel shows the result of GST-pulldown for BCA2, probed for myc-hHR23a. Whole cell GST blots are shown at 2 different exposures due to the differences in expression between GST-tagged BCA2 variants or GST alone, where the expression is substantially higher, and thus required a lower exposure. The difference in hHR23a binding is not a result of hHR23a expression, as lanes show equal loading for hHR23a and tubulin. [**C**] BCA2 binds to GST-tagged 14-3-3σ from bacterial cell lysates. Purified recombinant wild-type BCA2 (lane 2) as well as the RING (lane 8) mutant (mt) bind to 14-3-3σ (even lanes) but not the GST- alone protein (odd lanes). [**D**] Immunoblots of lysates from HEK293T cells which were co-transfected with FLAG-tagged BCA2 along with Xpress-tagged 14-3-3σ. BCA2 was introduced at 1 μg, 2 μg or 3 μg of plasmid DNA, while 14-3-3σ vector concentration was kept constant. The top panel shows increasing concentration of BCA2, the middle panel indicates a progressive decrease in expression of 14-3-3σ. β-Actin levels indicate decreasing 14-3-3σ is not an artefact of mis-loading (bottom part). [**E**] Immunoblots of lysates from HEK293T cells which were co-transfected with GST-tagged BCA2 along with myc/his-tagged hHR23A. BCA2 was introduced to the system in an increasing concentration, 1 μg, 2 μg or 3 μg, while the concentration of hHR23a vector was kept constant. The top panel shows increasing concentration of BCA2, the middle panel does not show any decrease in the expression of hHR23a, indicating there is no degradation. β-Tubulin levels indicate that the blot is equally loaded (third panel).

BCA2 contains three domains, the amino-terminal BCA2 Zinc-Finger (BZF) domain, the AKT phosphorylation domain, and the carboxy-terminal RING H2 domain (Figure 1A) [7,8]. BCA2's RING domain confers autoubiquitination activity, consistent with other E3 ubiquitin ligases such as RING proteins MDM2 and SIAH1 [2,9,10]. Touted as the "kiss of death" for proteins, ubiquitin is a highly conserved, 7 kDa protein modifier which targets proteins for proteasomal degradation. Ubiquitin conjugation to target proteins involves a number of well-coordinated steps, catalyzed by three enzyme types [11-14]. "Ubiquitination" has long had a negative connotation and in the past has been solely associated with the proteasome system. A staggering majority of enzymes that make up the UPS are particularly susceptible and seemingly promiscuously degraded not only through the actions of another ubiquitin ligase, *trans*-ubiquitination, but also through self-catalyzed ubiquitination. Recently, a review by de Bie and Ciechanover [15] discussed the mechanisms of regulation for E3 ligases. Both RING- and HECT-type ubiquitin ligases undergo various modifications and have multiple mechanisms that act to stabilize and/or activate these dynamic enzymes. Included in E3 modulation are substrate binding, phosphorylation and other post-translation modifications such as auto- or *trans*-ubiquitination for both proteolytic and non-proteolytic fates [15].

The wild-type BCA2 protein is unstable due to its autoubiquitination activity mediated by its RING domain. Substantial protein degradation has been shown in vivo and in vitro for the wild-type protein; however ligase-dead BCA2 variants showed no signs of degradation [2,7]. We and other have previously investigated partner of BCA2. Proteins shown to directly interact with BCA2 are Rab7, tetherin, ubiquitin and UBC9 [7,16-18]. In this study we identified additional BCA2 binding proteins. Ten new BCA2 interacting proteins were identified, two of which were hHR23a and 14-3-3σ. These proteins were chosen for further study as hH23Ra is a known chaperone in the ubiquitin-proteasome system (UPS) [19]. While 14-3-3σ, a multifunctional adaptor has been shown to have a role in many cancers including breast cancer [20]. The effects of hHR23a and 14-3-3σ on the stability and the autoubiquitination activity of BCA2, as well as co-expression of BCA2 and partner proteins in breast cancer were examined.

## Methods

### Antibodies

For Immunoblotting, the mouse monoclonal anti-FLAG (M2), anti-GST-2, anti-HA (HA-7), and anti-β-actin (AC-15) antibodies were purchased from Sigma-Aldrich Corp, (St. Louis, MO, USA). The monoclonal anti-polyhistidine (AD1.1.10) antibody was from R&D Systems (Minneapolis, MN, USA). The monoclonal anti-c-Myc (9E10) antibody was from Santa Cruz Biotechnology (Santa Cruz, CA, USA). Monoclonal anti-XPRESS antibody and mouse anti-β-Tubulin antibody were purchased from Invitrogen (Carlsbad, CA, USA). Monoclonal antibodies specific to hHR23a (ab55725) and 14-3-3σ (1.N.6) were purchased from abcam (Cambridge, MA, USA). Horseradish peroxidase-conjugated antimouse IgG secondary and Horseradish peroxidase-conjugated antimouse IgG secondary antibodies were from Promega (Madison, WI, USA).

For immunofluorescence (IF), primary antibodies for BCA2 and 14-3-3σ were used at 1 in 100 dilution, while hHR23a was used at 1 in 50 dilution. Secondary antibodies for IF, FITC-AffiniPure Goat Anti-Mouse IgG (H+L) and Cy3-AffiniPure Goat Anti-Rabbit IgG (H+L), were from Jackson ImmunoResearch Laboratories Inc. (West Grove, PA, USA).

For immunohistochemistry (IHC), all primary antibodies were used at dilutions of 1 in 100. Secondary antibodies for IHC were from VECTOR laboratories (Burlington, ON, Canada), biotinylated anti-mouse IgG (H+L) or biotinylated anti-rabbit IgG (H+L) were used at a dilution of 1-200.

### Vectors and cloning

The hHR23a construct was created by PCR amplifying from a cDNA library using the forward primer 5'-GACTGGATCCATGGCCGTCACCATCACGCTC-3' which contains a BamHI restriction site, and the reverse primer 5'-GACTCTCGAGCGCTCGTCATCAAAGTTCTCACTC-3' containing an XhoI restriction site. PCR fragments and pSG5-myc/his backbone were digested with BamHI and XhoI and transformed into cloning cells. 14-3-3σ construct was created by cutting the 14-3-3σ gene via restriction digest from a pre-existing bacterial expression vector pET100 containing the XPRESS-expression tag. AKT vectors were generously donated to our laboratory [8]. FLAG BCA2 vectors are as described [7]. GST-tagged BCA2 constructs are in the pEBG backbone, and were cut in from the pCMV constructs previously created [7].

### Bacteria- and yeast -II-hybrid screening

Bacteria-II hybrid screening followed Stratagene BacterioMatch protocol. BCA2 bait vector was created by cutting FLAG-tagged BCA2 from pCMV-Tag2B vector (previously described [7]) with restriction enzymes NotI and XHoI and ligating into the pBT vector. cDNA target library, in the pTRG vector, was derived from human breast cells pooled from 10 donors between 33 to 88 years. The selection of positive transformants was mediated by X-Gal indicator plates and blue and white screening.

Bait vector for yeast-II-hybrid screening was created by cutting the BCA2 construct from BCA2-containing pCMV-Tag-2B [7] with BamHI and SalI and ligating into the pSOS vector. Target library in pMyr, containing a plasma membrane localizing myristolation signal, was created from HeLa cells according to the CytoTrap protocol (Agilent Technologies Inc., Wilmington, DE, USA). Selection of positive clones was mediated through the temperature sensitive activation of the RAS-pathway by recruitment of SOS-tagged bait which allows yeast growth.

### Cell culture, transfection and protein lysis

Breast carcinoma cell lines BT474, MCF7, MDA MB 231, MDA MB 435, MDA MB 436 and MDA MB 453 as well as HEK293T cells were grown in DMEM with 10% FBS (Invitrogen) and 1% penicillin/streptomycin (Invitrogen), at 37°C and 5.0% CO_2_. For transfection in HEK293T cells, cells were seeded to 6-well plates at an appropriate concentration. Cells were allowed to settle overnight prior to transfection. Transfections were performed using Lipofectamine 2000 transfection reagent (Invitrogen) according to the manufacturer's instructions.

Cells were washed and harvested in PBS, then lysed using lysis buffer containing 1% Nonidet P40 with proteinase inhibitors (1 mg/ml pepstatin A, 5 mg/ml leupeptin, 0.5 M EDTA, 1.6 mg/ml aprotinin, 100 mM PMSF). Protein quantitation was done by Bradford assay (BioRad, Hercules, CA, USA).

### GST-pulldown and co-immunoprecipitations

Mammalian cells were lysed in NP-40 Lysis buffers without EDTA, for bacterial cells; lysis in PBS occurred by sonication using 3 rounds of 5 × 1 seconds pulses on ice. Immunoprecipitations were performed using 25 μl of sepharose beads (GE Healthcare, Pittsburgh, PA) per sample. Whole cell lysates (WCL) were added to beads at protein concentrations between 0.3 mg to 1 mg for mammalian cell lysates. For bacterial lysates, protein concentration was determined by western blot prior to pulldown.

For co-immunoprecipitations (Co-IP), 250-500 μg of cell lysates were used per sample. Lysates (3-5 ug/sample) were incubated with antibodies overnight at 4°C with gentle agitation. Protein A/G beads (Santa Cruz) (20 ul/tube) were aliquoted to microcentrifuge tubes, followed by the addition of antibody conjugated lysates.

For both GST-pulldowns and Co-IPs, beads and lysates were incubated at 4°C for 1 hour with constant agitation. Washed sample were boiled for 10 minutes at 95°C in 1 × Sodium Dodecylsulfate (SDS) loading buffer.

### SDS-PAGE and western blotting

Proteins (25-50 ug) were resolved on 12% SDS denaturing- polyacrylamide gels and transferred to nitrocellulose membranes (BioRad). Following transfer membranes are transiently stained with 1 × Ponseau stain to visualize proteins loading. Blots were blocked in 5% skim-milk powder in TBST (TBS buffer containing 1% Tween) for 1 hour at room-temperature, incubated with primary antibodies overnight at 4°C, washed in TBST, and then incubated with secondary antibodies, HRP-conjugated anti-mouse or anti-rabbit (Promega) secondary antibodies, for 1 hour at room-temperature. Following washes in TBST, proteins were visualized by addition of Chemi-luminescence reagent, and exposed to X-Ray film (VWR, Radnor, PA, USA). Blots were scanned and quantified using ImageJ ver. 1.44 [21].

### Immunofluorescence

Immunofluorescence was performed on cells grown on Lab-Tek Chamber slides (Fisher Scientific, Rochester NY, USA). Cells were fixed in 4% paraformaldehyde (PFA) and blocked using normal goat serum (Jackson ImmnoResearch Laboratories) for a minimum of 1 hour at room temperature. Sections were incubated with primary antibodies overnight at 4°C, in a dark container. The next morning slides were washed then incubated with fluorescence-conjugated secondary antibodies for an hour at room temperature in a dark container. After washing, slides were mounted using with DAPI (Vectashield mounting medium, VECTOR Laboratories Inc, Burlingame CA, USA), edges of the coverslip were then sealed.

### Tissue microarrays

Tissue microarrays were generously donated by the Department of Anatomic Pathology at Sunnybrook Health Sciences Centre. In accordance with ethics protocols, specimens were stripped of all patient identifiers and blinded. Arrays were assembled using paraffin-embedded breast cancer tissues which had been previously analyzed for tumor areas by pathologists. Two master blocks, 105 cases total, were used to produce serial sections for staining. Blocks contained either 50 or 55 cases of 1.0 mm cores in triplicate. Slide sections were cut to a thickness of 5 μm, and mounted on PSA 4 × Slides (Leica Biosystems Richmond Inc, Richmon IL, USA), followed by drying at 60°C for 1 hour.

### Immunohistochemistry

Immunohistochemistry was performed on paraffin-embedded tumor sections. Slides were de-waxed and re-hydrated by sequential baths in Xylene, ethanol and PBS. Antigen unmasking occurred though pressure cooking slides in citrate buffer (pH 6.0). Slides were processed with H_2_O_2 _for 30 minutes and were blocked in normal goat serum (NGS) (Jackson ImmnoResearch Laboratories), for 1 hour at room temperature. Slides were incubated with primary antibodies overnight at 4°C. Secondary biotinylated antibodies were applied for 1 hour at room temperature. ABC complex (Vectastain ABC kit, VECTOR Laboratories) was applied to sections for 30 minutes at room temperature, followed by DAB (Peroxidase Substrate Kit- DAB, VECTOR Laboratories). Slides were treated with DAB for approximately 2-3 minutes then counter-stained with hemotoxylin for 30-45 seconds. Slides were dehydrated prior to mounting using Cytoseal XYL (VWR).

## Results

### Binding partners of BCA2

Bacteriomatch bacterial-II-hybrid and Cytotrap yeast-II-hybrid (Agilent) techniques were used to identify partner proteins of BCA2. BCA2, cloned into pBT, for bacterial screening or pSOS, for yeast screening, was used as bait to isolate potential partners from a pTRG breast tissue cDNA library or a HeLa pMyr target library, respectively. Novel positive clones identified by this both yeast and bacteria screens are listed in Table 1. Proteins of interest were those which affect the stability of their partner proteins. Both hHR23a, a component of the UPS, and 14-3-3σ, which binds partner proteins through AKT phosphorylation sites, were selected for further study. The interaction of BCA2 with ha-c-Ras, inidicated that the yeast screening system worked optimally and serves as an internal positive control, as Ras is a down-stream component of SOS, and is an anticipated interaction.

**Table 1:** Putative binding partners of BCA2

Gene	Accession Number	Function
*14-3-3σ	AF029082	Proliferation, signal transduction and apoptosis

*Human homolog of Rad23 variant A	DBJ|BAD9250.1	DNA Repair, chaperone

Atrophin-1	U23851	Transcription factor

CDC2-related protein kinase 10 (CDK10)	L33264	Cell proliferation

c-Ha-ras-1	PRF|090402A	Cell signalling

Cystatin-C	BC013083	Inhibitor of cysteine proteases

Dedicator of cytokinesis 4 (DOCK4)	gb|AAI17689.1	Regulates cell-cell adhesion

FOLR3 protein	gb|AAH30285.1	Internalization of folic acid

Microfibril-associated glycoprotein 4	BC022666	Cell adhesion & cell-cell interactions

SNC73 protein	AF067420	Colorectal cancer marker

### BCA2 binds hHR23a and 14-3-3σ

To validate BCA2 interactions with putative partner proteins, GST-tagged BCA2, along with BZF, S132, 133A and RING mutants as well as the GST control were co-transfected into HEK293T cells with myc-hHR23a. Cell lysates subjected to GST-pulldowns, interactions were visualized though Western blotting using myc antibodies. As shown in Figure 1B, hHR23a was bound by all tested mutants of BCA2 as well as wild-type protein (lanes 1-4, top panel), but not by the GST control (lane 5, top panel). This indicated a specific interaction of hHR23a with BCA2. hHR23a protein bands in BZF and RING mutants lanes (Figure 1B, lanes 2 and 4, top panel) appear to have higher intensity than BCA2 and S132, 133A mutant lanes (Figure 1B, lanes 1 and 3, top panel), this observation likely is to due to the elevated cellular expression of the RING and BZF mutants of BCA2.

BCA2 interacted with 14-3-3σ by GST-pull-down assay. Recombinant Flag-BCA2 was incubated with sepharose-immobilized GST control or GST-14-3-3σ. Immunoblot shown in Figure 1C, indicated that the wild-type BCA2 and BCA2 RING mutant bound 14-3-3σ specifically (lanes 2 and 8). There was no interaction with the GST control protein (Figure 1C, lanes 1 and 7). Moreover, it appeared that the disruption of the BZF domain or the AKT domain abolished binding with 14-3-3σ. This suggested that that these domains are important to BCA2 interaction with 14-3-3σ (Figure 1C, lanes 3-6).

### 14-3-3σ but not hHR23a is a substrate of BCA2

Both hHR23a and 14-3-3σ were investigated as potential substrates of BCA2 E3 activity. An increasing amount of Flag-BCA2 (1.0 μg-3.0 μg plasmid DNA) was co-transfected into HEK293T cells along with a constant concentration of 14-3-3σ (1.0 μg) (Figure 1D). Samples were analyzed by Western blotting, Figure 1D showed that increased amount of expressed BCA2 inversely correlated with the expression of 14-3-3σ protein. This decreased 14-3-3σ expression in relation to BCA2 expression suggested that 14-3-3σ may be signaled for degradation by BCA2 ligase activity.

To investigate the ability of BCA2 to regulate hHR23a, we examined whether hHR23a would be degraded upon interaction with BCA2 (Figure 1E). A stable concentration of hHR23a (1.0 μg) plasmid DNA and an increasing concentration of BCA2 (1.0 μg-3.0 μg) plasmid DNA were co-transfected into HEK293T cells and visualized by immunoblotting. Figure 1E showed the expression of hHR23a was not decreased in relation to increasing BCA2 expression. This suggested that hHR23a is not a substrate for BCA2-mediated degradation. However, we noticed that hHR23a expression is more robust where BCA2 protein levels were elevated. This indicated that while not poly-ubiquitinated, hHR23a may be mono- or multi-ubiquitinated by BCA2.

### 14-3-3σ and hHR23a are co-expressed in BCA2 positive breast cancer cell lines

**Figure 2:**
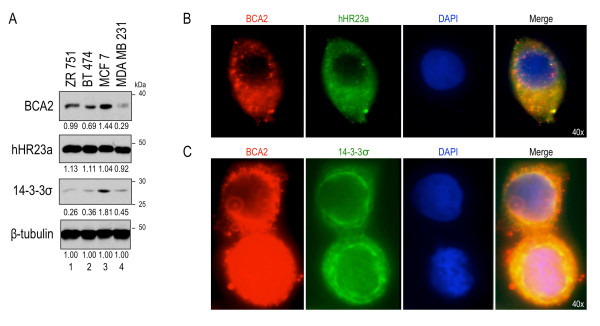
**Endogenous expression and localization of BCA2 and partner proteins**. [**A**] Western blot comparing expressed protein levels of BCA2, hHR23a, 14-3-3σ and β-Tubulin in ER-positive and ER-Negative cell lines. Normalized relative intensities of each protein are displayed below their respective blots. Microscopy images, taken at 100 × magnification, of endogenous IF staining for [**B**] BCA2 and hHR23a or [**C**] BCA2 and 14-3-3σ, tagged with either secondary antibody conjugated to FITC-dye or Cy3, in MCF7 breast cancer cells. The last panel shows the merged image and co-localization of the proteins.

Whole cell lysates from a panel of breast cancer cell lines were analyzed via Western blotting. Blots were probed to investigate the endogenous expression of hHR23a, 14-3-3σ and BCA2. BCA2 has been shown to be transcriptionally up-regulated in ER-positive mammary epithelial cell lines [2,16], protein expression levels shown in Figure 2A are consistent with those observations [2,16]. ER-positive cell lines ZR751, BT474 and MCF7 (Figure 2A, lanes 1-3) have elevated expression of the BCA2 protein compared with ER-negative cell line MDA MB 231 (Figure 2A, lane 4). Expression of hHR23a was marginally increased in ER positive cells as determined by densitometry. Analysis further showed that BCA2 was upregulated in cells expressing high amounts of 14-3-3σ. The expression of 14-3-3σ was variable between cell lines, likely due the epigenetic regulation of this protein in cancer cells.

From the Western blots of breast cancer cell lines, we observed that BCA2, 14-3-3σ and hHR23a are expressed in the ER-positive cell line MCF7 (Figure 2A). The cellular localization of these proteins was analyzed through immunofluorescent staining of endogenous proteins (Figure 2B and 2C). hHR23a and BCA2 were observed in the cytoplasmic and nuclear compartments (not shown). In the cytoplasm BCA2 showed a punctate staining pattern (Figure 2B, first panel), while hHR23a a showed a more diffuse staining pattern (Figure 2B, second panel). Merged images indicated a large area of co-expression of BCA2 and hHR23a MCF7 cells (Figure 2B, last panel). Unlike BCA2, 14-3-3σ exclusively localized to the cytoplasm (Figure 2C, middle panel). In the cytoplasmic compartment, significant overlay was noted between 14-3-3σ and BCA2 (Figure 2C, bottom panel). These data confirmed that not only are these proteins co-expressed in MCF7 cells, but also that BCA2 co-localized with both hHR23a and 14-3-3σ.

### BCA2 and hHR23a expression correlates in breast cancer tissues

**Figure 3:**
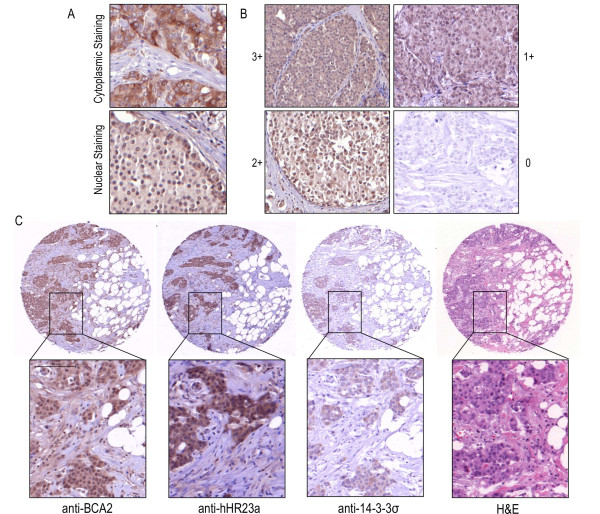
**Immunohistochemistry of breast cancer TMA (n = 105)**. [**A**] An example of how tumors were scored for protein localization. The top panel is staining positively in the cytoplasm with no nuclear staining, the bottom panel shows a tissue which is staining weakly in the cytoplasm and stongly in the nuclear compartment. [**B**] Shows examples of the criteria for staining intensity, where 3+ (top left) has the strongest staining/most intense and 1+ (top right) which has the weakest staining/least intense. A tissue staining negatively (0), is also shown (bottom right). [**C**] Shows a typical staing pattern for a serial section of the same tumor at 5 × magnification and 20 × magnification for BCA2, hHR23a and 14-3-3σ immunostaining, aswell as H&E staining.

The prevalence of co-expression of BCA2 and either hHR23a or 14-3-3σ was evaluated in multiple breast cancer cases. Serial sections of two breast cancer tissue microarrays (TMA) totalling 105 case were were assessed. TMA immunostaining was visualized with DAB following probing with antibodies against BCA2, hHR23a and 14-3-3σ. Sections were scored for percentage of cells staining in the cytoplasmic or nuclear compartments of cells. An average of the triplicate punches was used to score each case. Cells were designated as having nuclear staining, if the nuclear compartment was significantly darker than the surrounding cytoplasmic staining, to rule out mis-scoring due to cytoplasm overlaying the nucleus. Percent abundance of staining as it pertained to each cellular compartment was defined by the number of tumor cells which expressed the proteins in question in each location, independent of expression of proteins in the other compartments (examples of staining are shown in Figure 3A). Sections were further scored for the intensity of DAB staining, and were classified as either 3+, 2+, 1+ or 0 (Figure 3B), corresponding to strong, medium, weak or negative staining (staining verified by J.Z). Cells were considered to have low staining if intensity was scored at 0 or 1+ or if 10% or less of tumor cells were staining. Cores were considered to be staining highly if more than 10% of tumor cells were staining at an intensity of 2+ or 3+.

Of the 105 tumors stained with the BCA2 antibody, 97.1% stained positively in the cytoplasm and 60% stained positively in the nucleus. 93.3% percent of tissues stained positive for hHR23a in the cytoplasm and 54.3% of tumor stained positively in the nucleus. 14-3-3σ was observed as exclusively staining in the cytoplasm of tumor cells, and was scored as positively staining in 61.9% of tissues. Co-expression of these proteins was analyzed based on intensity and percent of positive tumor cells. Expression was again broken down by nuclear and cytoplasmic localization and analyzed with Fisher's exact t-Test. Cytoplasmic expression of BCA2 was found to correlate significantly (P = 0.0113) with cytoplasmic hHR23a expression, where BCA2 is highly expressed in tissues which highly expressed hHR23a. BCA2 and 14-3-3σ did not correlate significantly in cytoplasmic expression (P = 0.0804), however, the numbers trended towards low levels of 14-3-3σ expression in the presence of high BCA2 expression, 41.9% of 105 tumors. More significantly than cytoplasmic correlation between hHR23a, was the nuclear co-expression. Tissues with low BCA2 also appeared to have low hHR23a, and vice versa, tissues with high BCA2 expression also had high hHR23a expression (P = < 0.0001).

BCA2 expression was further analyzed in terms of the clinical variables Grade, ER status, PR status and HER2 status (Table 2). Grade was the only clinical variable which showed a statistically significant correlation with BCA2 expression (P = 0.0297). High levels of BCA2 expression was correlated with a diagnosis of grade 2 breast cancer. Our study did not show any statistically significant correlation estrogen receptor or progesterone receptor in either the cytoplasm or the nucleus. However, in the context of both ER and PR, BCA2 was more likely to be high in positive cells, 50 cases and 39 cases out of 103 respectively. Localization of hHR23a was also compared to clinical variables (Table 2). Cytoplasmic hHR23a correlated with positive ER expression (P = 0.0181). Moreover, while not statistically significant, high cytoplasmic hHR23a trends towards negative nodal status (P = 0.0585).

**Table 2:** Expression of BCA2 and hHR23a in breast cancer cases

	Cytoplasmic BCA2					Nuclear BCA2					Cytoplasmic hHR23a					Nuclear hHR23a				
**Variable**	**Low (%)**		**High (%)**		**P-value**	**Low (%)**		**High (%)**		**P-value**	**Low (%)**		**High (%)**		**P-value**	**Low (%)**		**High (%)**		**P-value**

ER																				

Positive	27	(34.2)	52	(65.8)	0.8053	29	(36.7)	50	(63.3)	0.4772	27	(34.2)	52	(65.8)	*0.0181	39	(50.0)	39	(50.0)	0.4947

Negative	7	(29.2)	17	(70.8)		11	(45.8)	13	(54.2)		2	(8.3)	22	(91.7)		10	(41.7)	14	(58.3)	

PR																				

Positive	17	(31.3)	39	(68.7)	0.6743	17	(30.4)	39	(69.6)	0.0686	19	(33.9)	37	(66.1)	0.1897	26	(47.3)	29	(52.7)	1.000

Negative	17	(36.1)	30	(63.9)		23	(48.9)	24	(51.1)		10	(21.3)	37	(78.7)		23	(48.9)	24	(51.1)	

HER2																				

Positive	6	(35.3)	11	(64.7)	1.000	8	(47.1)	9	(52.9)	0.5878	5	(29.4)	12	(70.6)	1.000	10	(58.8)	7	(41.2)	0.4292

Negative	28	(32.9)	57	(67.1)		32	(37.6)	53	(62.4)		23	(27.1)	62	(72.9)		39	(46.4)	45	(53.6)	

Grade																				

1	2	(22.2)	7	(77.8)	0.5501	4	(44.4)	5	(55.6)	* 0.0297	3	(33.3)	6	(66.7)	0.2730	5	(55.6)	4	(44.4)	0.4142

2	18	(38.3)	29	(61.7)		12	(25.5)	35	(74.5)		14	(29.8)	33	(70.2)		19	(41.3)	27	(58.7)	

3	14	(30.4)	32	(69.6)		24	(52.5)	22	(47.8)		12	(26.1)	34	(73.9)		25	(54.3)	21	(45.7)	

Nodal																				

Positive	11	(32.4)	23	(67.6)	1.000	10	(29.4)	24	(70.6)	0.1952	14	(41.2)	20	(58.8)	0.0585	17	(51.5)	16	(48.5)	0.6649

Negative	20	(33.3)	40	(66.7)		26	(43.3)	34	(56.7)		13	(21.7)	47	(78.3)		27	(45.0)	33	(55.0)	

* Indicates a correlation which is statistically significant by Fisher's exact t-test or *χ*^2 ^test

### BCA2 is unstable in the presence of E2s of the UbcH5 family

**Figure 4:**
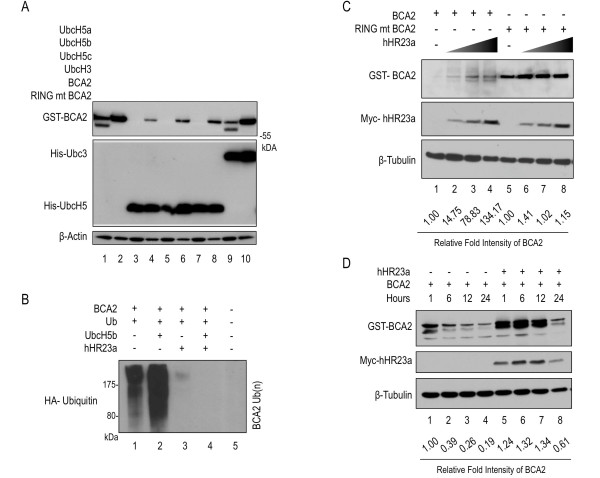
**hHR23a rescues and stabilizes BCA2 from UbcH5 mediated poly-ubiquitination**. [**A**] Immunoblots of lysates from HEK293T cells cotransfected with GST-tagged BCA2 (GST-BCA2) and BCA2 with the RING domain mutation C228/231A (GST-BCA2-RING mt) and/or expression vectors for His-tagged Ubc3 and UbcH5a/b/c as indicated. [**B**] Western blots of HEK293T cells which were co-transfected with stable amounts of either GST-tagged wild-type BCA2 or GST-BCA2 RING mt along with an increasing amount of myc/his-hHR23a. Relative intensity of BCA2 normalized to β-Tubulin is given below the blot as fold change compared to lanes 1 and 5 respectively. [**C**] Immunoblot showing lysates of in vivo ubiquitination assay probed with anit-HA following immunoprecipitation of Flag-BCA2 with anti-FLAG antibodies. HEK293T cells were co-transfected with HA-ubiquitin, and FLAG-BCA2 along with either myc/his-UbcH5b or myc/his-hHR23a or both as indicated above lanes. [**D**] Immunoblot of a half-life assay of HEK293T cells transfected with GST-BCA2 with and without myc-hHR23a. Cells were treated over a 24 hours time course with cyclohexamide. Relative intensity of BCA2 normalized to β-Tubulin is given below the blot as fold change.

BCA2 has a very strong autoubiquitination activity in the presence of the E2 UbcH5b, which resulted in its rapid degradation [2]. To determine whether other E2s are involved in BCA2 degradation, HEK293T cells were co-transfected with expression vectors for E2 enzymes UbcH5a, UbcH5b, UbcH5c, or Ubc3 as well as GST-BCA2 or ligase-dead GST-BCA2 RING mutant. Western blot analysis showed that in the absence of E2, both the wild-type BCA2 and RING mutant are visible when probed with anti-GST antibodies (Figure 4A lanes 1 and 2, top panel). However, in the presence of enzymes of the UbcH5 family, wild-type BCA2 was degraded and therefore undetectable by immunoblot (Figure 4A, lanes 3-8, top panel). The RING-mutant expression was maintained under these conditions due to its abrogated autoubiquitination activity. Both BCA2 and RING mutant were strongly expressed in the presence of Ubc3 (Figure 4A, lanes 9 and 10, top panel). This suggested that Ubc3 had no effect on the degradation of BCA2 (Figure 4A, lanes 9 and 10, top panel).

### hHR23a inhibits BCA2 autoubiquitination activity and stabilizes BCA2

In its role as an ubiquitin receptor, hHR23a is known to prevent the formation of polyubiquitin chains, and thus inhibits degradation of target proteins [22,23]. The effect of hHR23a on BCA2 polyubiquitin chain formation was examined through Ubiquitination assays. HEK293T cells were co-transfected with BCA2 and Ubiquitin expression vectors; along with hHR23a and/or UbcH5b. Following immunoprecipitation (IP) of BCA2, we observed intense, high molecular weight ubiquitination smears which started at ~75 kDa (Figure 4B). These smears are characteristic of BCA2 autoubiquitination [7]. Ubiquitin-laddering was present when BCA2 was expressed alone (Figure 4B lanes 1 and 2), or with UbcH5b where BCA2 auto-ubiquitination increased. However, when hHR23a was co-expressed in the system, the BCA2 ubiquitination smear dramatically diminished or was undetectable (Figure 4B, lanes 3 and 4). This suggested that hHR23a acts as an inhibitor of BCA2 autoubiquitination ability by abrogating multi-ubiquitin chain elongation.

To further investigate the outcome of the interaction between BCA2 and hHR23a, HEK293T cells were co-transfected with a constant concentration of BCA2 or BCA2 RING mutant and an increasing concentration of hHR23a plasmid DNA (0 μg-2 μg) (Figure 4C). Western blots were analyzed for relative intensity of BCA2 protein levels, normalized to β-Tubulin. We observed a positive correlation between elevated BCA2 expression and increased hHR23a concentration. Up to 130-fold increase was evident with 2 μg of hHR23A, as compared with no hHR23A expression. There was no significant change observed for BCA2 RING mutant protein expression in the presence of hHR23a. This indicated that hHR23a stabilized BCA2 in a dose dependent manner.

BCA2 half-life expires rapidly in vivo [2]. Figure 4B and 4C showed that hHR23a increased the stability of BCA2 through the inhibition autoubiquitination. Stabilization of BCA2 by hHR23a was further examined through half-life assays, HEK293T cells were co-transfected with hHR23a and BCA2 and protein extracts were harvested at 1, 6, 12 and 24 hours time points following addition of CHX or DMSO. Protein expression was and compared via western blot (Figure 4D). BCA2 protein level diminished more rapidly in the absence hHR23a in CHX-treated cells. Conditions where BCA2 was co-expressed with hHR23a exhibited less degradation of BCA2 over time. DMSO control treatments (data not shown) indicated that the effects observed was a result hHR23a. These data suggested that BCA2 half-life was increased upon interaction with hHR23a.

### 14-3-3σ binds BCA2 through interaction with its phosphorylated AKT domain

**Figure 5:**
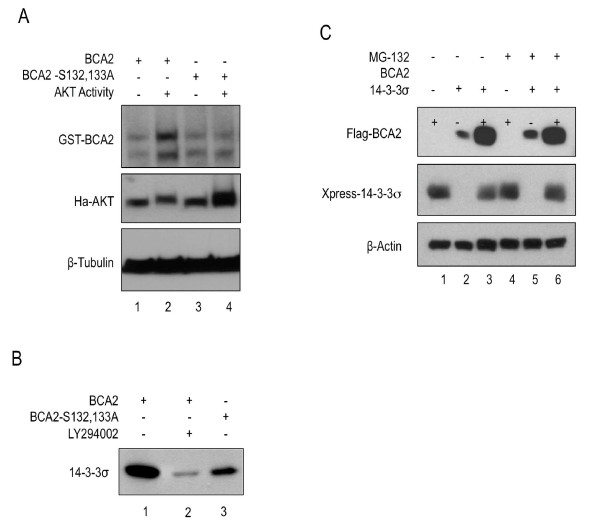
**AKT mediated phosphorylation leads to an increase in BCA2 stability through 14-3-3σ interaction**. [**A**] Immunoblot of equal amounts of lysates of HEK293T cells co-transfected with wild-type GST-BCA2 or GST-BCA2 S132, 133A along with either dominant negative or constitutively active AKT. [**B**] Co-immunoprecipitation of wild-type BCA2 and 14-3-3σ in the presence (lane2) or absence (lanes 1, 3) of AKT inhibitor LY294002 and co-immunoprecipitation of S132, 133A BCA2 and 14-3-3σ. [**C**] Western blot showing the results of a stability assay in the presence of absence of MG-132 on lysates of HEK293T cells which had been tranfected with either wild-type FLAG-BCA2, Xpress-14-3-3σ or both.

Protein phosphorylation is arguably the most common covalent modification of proteins, and induces changes to conformation and stability [24]. BCA2 was shown to be phosphorylated in the AKT domain [8]. To investigate the role of phosphorylation on BCA2 stability, HEK293T cells were co-transfected with a constitutively active variant of AKT or a kinase-dead AKT mutant, along with FLAG-tagged BCA2 (Figure 5A, lanes 1 and 2) or the S132, 133A mutant which abolished AKT phosphorylation (Figure 5A, lanes 3 and 4). Figure 5A showed that wild-type BCA2 is stabilized by the addition of constitutively active AKT (lane 2) compared with BCA2 kinase-dead AKT (lanes 1 and 3). Furthermore, there was no change in the expression of the BCA2 S132, 133A mutant [7]. This indicated that post-translational modification of BCA2 is necessary to its regulation.

*In silico *analysis of BCA2 revealed that AKT domain contains 14-3-3 binding sequences. Immunoprecipitations (Figure 5B) of lysates co-expressing BCA2 and 14-3-3σ indicated that 14-3-3σ co-immunoprecipitated with the wild-type BCA2 with greater affinity than with the S132, 133A mutant (lanes 1 and 3). Moreover, when treated with AKT-kinase inhibitor LY294002 (Figure 5B, lane 2), 14-3-3σ bound to wild-type BCA2 with lesser affinity compare to untreated wild-type BCA2 lysate or the untreated S132, 133A lysate. Together these data suggested that the phosphorylation of BCA2 by AKT was important to partner interaction.

### BCA2 is stabilized by substrate interactions with 14-3-3σ

Some E3 ligases have been known to be stabilized by substrate interaction. We assayed the ability of 14-3-3σ to affect the stability of the BCA2 protein. HEK293T cells co-expressing Xpress-14-3-3σ and wild-type Flag-BCA2 were incubated with protein degradation inhibitor MG-132 (Figure 5C) or DMSO control. Lysates in which wild-type BCA2 was expressed alone showed decreased levels of BCA2 protein though immunobloting compared to samples where BCA2 was co-expressed with 14-3-3σ (Figure 5C). This was observed both in the presence and the absence of MG-132. These data suggested that BCA2 was stabilized more by expression of partner proteins than by inhibition of protein degadation. Moreover, the experiment further indicated that 14-3-3σ is a substrate for BCA2 as 14-3-3σ expression decreased upon the expression of BCA2.

## Discussion

An integral component of understanding E3 ligase function is identifying the enzyme's cognate substrate(s) and/or binding proteins. Previous binding partners of BCA2 included Rab7, isolated through yeast-II-hybrid screening [17], tetherin, which was found though brute-force GST-pulldowns [18], and ubiquitin and UBC9 from bacteria-II- hybrid screening [7,16]. Bacteria and yeast screening systems were used in this study to identify additional potential binding partners of BCA2 (Table 1). Of the potential partners found, 14-3-3σ and hHR23a were chosen for further investigation in the context of BCA2 activity and expression. Through binding experiments we confirmed that the BCA2 protein bound both to 14-3-3σ and hHR23a (Figure 1B and 1C). hHR23a and BCA2 were co-expressed in a mammalian system, while 14-3-3σ was expressed in a bacterial system and incubated with recombinant purified BCA2. The expression levels of wild-type BCA2 and the S132, 133A mutant in HEK293T cells were much lower than those of the RING-mutant and BZF mutant, which is a possible confounding factor in determining domains necessary for binding hHR23a. The overexpression of BZF and RING mutants likely resulted from steric hinderance of BCA2 autoubiquitination activity from the GST-expression tag. Both the wild-type BCA2 protein and the RING mutant, but not the BZF or S132, 133A mutants of BCA2 were pulled down by 14-3-3σ, indicating that sequences in the BZF domain or AKT domain may be necessary for interaction.

Protein interaction was confirmed in an endogenous system. Breast cancer cell lines were probed for expression of hHR23a and 14-3-3σ, along with BCA2 (Figure 2A). BCA2 has previously been reported as co-expressing with estrogen receptor [2,16], thus high expression was expected in ER-positive cell lines ZR751, BT474 and MCF7, while lower expression was expected in the ER-negative cell line MDA MB 231. Our findings were consistent with these expectations. hHR23a was expressed in all cell lines, but had slightly higher expression in the ER-positive cell lines, which correlated with the co-expression of hHR23a and ER in breast cancer TMAs. Immunofluorescence studies confirmed that endogenous proteins co-localized.

Protein expression was further investigated in the context of breast cancer analyzed though IHC. Tumor cores which stained highly positive for BCA2 also stained highly positive for hHR23a in both the nucleus and the cytoplasm. This correlation was determined to be significant by Fisher's Exact T-Test. From this we inferred that not do these proteins interact, but they are co-expressed endogenously in the same cells and tissues. While not statistically significant, in the tumors BCA2 and 14-3-3σ trended towards an inverse relationship in expression. The majority of samples which stained highly for BCA2 were scored as having low expression of 14-3-3σ.

A problem which emerged over the course of studying BCA2, was the unstable nature of the protein due to intrinsic autoubiquitination activity. Initial investigations of BCA2 stability, found that degradation via autoubiquitination was equally possible with all UbcH5 family members, but not with other E2's such as the UbcH3 ubiquitin conjugating enzyme. Moreover, the presence of members of the UbcH5 family was responsible for the rapid and complete degradation of wild-type BCA2 protein in vitro and in vivo (Figure 4A) [2,7].

As a molecular chaperone, hHR23a regulates the lifespan of its binding partners in both agonisticly and antagonistically. In the proteasome system, hHR23a acts as a shuttle to the proteasome, by simultaneously binding both the target protein and the lid of the 26 s proteasomal subunit. In contrast to this function, hHR23a also regulates protein half-life through sequestering nascent ubiquitin moieties in the emerging polyubiquitin chain. Thereby preventing chain elongation and de-ubiquitinase activity and stabilizing the targeted protein [25]. This dual nature of hHR23a is largely due to the presence of its N-terminal Ubiquitn-Like domain (UBL) and two C-terminal Ubiquitin-Associating (UBA) domains [26]. The UBL domain may be a key component of hHR23a's interaction with BCA2. The significance of this interaction is such that it increases the stability of BCA2. Increased concentration of the partner protein correlated with the elevated levels of BCA2 protein (Figure 4C). We inferred that BCA2 was stabilized through the prevention of ubiquitin chain elongation by hHR23a (Figure 4B). This was consistent with reported behaviour of hHR23a as an ubiquitin receptor. These observations were strengthened since hHR23a was also shown to increase the half-life of BCA2 over a 24 hours time period (Figure 4D). hHR23a was not a substrate for BCA2-mediated degradation (Figure 1E). This does not exclude hHR23a mono- or multi-ubiquitination from being catalyzed by BCA2. Figure 4E showed an increased stability of hHR23a in the presence of high BCA2 expression, which may result from ubiquitin modification of hHR23a, which has been previously reported [22,27]. Taken together, data presented here argues that hHR23a presence may be necessary to modulate and regulate BCA2 in a cancer setting.

As well as through interaction with hHR23a, BCA2 was also increased through modification by AKT phosphorylation. Phosphorylation has far reaching consequences for the modified proteins, amoung them are conformational changes which effect substrate affinity and specificity [28,29]. It had been previously demonstrated that BCA2 was phosphorylated in the presence of AKT [8]. Here, we showed that BCA2 was more stable when co-expressed with constitutively active AKT as opposed to kinase-dead AKT. The S132, 133A mutant displayed no notable change in stability. This confirmed that these serine residues are the primary sites of AKT-mediated BCA2 phosphorylation. Surrounding S132 and S133 are two 14-3-3σ binding motifs. The 14-3-3σ motif R(S/X)XpSXP [30] is highly similar to the BCA2 protein sequence 130-RGSSRP-135 (Figure 1A). 14-3-3σ is a member of the ubiquitously expressed 14-3-3 family of proteins. The seven proteins of this family are highly conserved and integral to a number of important cellular activities. Specifically, 14-3-3σ is known to be a conspicuous regulator of cell cycle checkpoints, as well as being involved in multiple and diverse cellular pathways through interactions with its copious number of known ligands [20,31-33]. We demonstrated that phosphorylation was an integral element of the interaction between BCA2 and 14-3-3σ. In samples where the AKT domain of BCA2 was disrupted or where wild-type BCA2 was treated with AKT inhibitor, BCA2 was less apt to bind 14-3-3σ. However, since interaction between 14-3-3σ and BCA2-S132, 133A was not completely abolished, it is likely that a second functional AKT-phosphorylation site and 14-3-3σ binding site exists. We predicted this site to be in the adjacent sequence, 133-SRPDRSPS-140 (Figure 1A).

Similar to hHR23a, the outcome of interaction of BCA2 with 14-3-3σ was an increase in BCA2 stability. Presumptively, BCA2 is stabilized as 14-3-3σ sterically or conformationally prevents access of the RING finger domain to the target lysines (K26 and K32) in the BZF domain [7]. More than stabilizing BCA2, this interaction decreases the protein levels of 14-3-3σ (Figure 1D). This effect was not seen when the same experiment was repeated with hHR23a. hHR23a levels did not decrease with the increase in BCA2 (Figure 1E), suggesting that 14-3-3σ but not hHR23a is a substrate of BCA2. Other E3 ligases that have been demonstrated to be stabilized by the binding of their substrate include HOS (homologue of Slimb), where presence of substrate IκBα stabilized HOS turnover [34]. Another examples being Cdc 4 (cell division cycle 4), which binds ubiquitin to promote its own degradation in the absence of substrate, as the binding of ubiquitin and substrate are mutually exclusive events. However when substrate is present, Cdc4 is stabilized, suggesting that substrate concentration is a method of E3 regulation [35]. Moreover, the lack of considerable stabilization by the presence of a proteasome inhibitor alone, provides basis for the argument that BCA2 may not be solely degraded in a proteasome-mediated pathway, but may be regulated by other pathways, such as the lysosomal degradation pathway, similar to c-Cbl, which auto-regulates and is degraded via the lysosome [36-38].

Pharmacological pursuits in the area of cancer therapies have in the past been aimed at the non-specific components of the UPS. The most effective and promising of these therapeutics is Bortezomib (Velcade™), which inhibits the chymotryptic activity of the proteasome [39,40]. As the components of the UPS are arranged in a pyramidal way, with few E1 enzymes at the top and multiple and specific E3 ligases at the base, they make attractive and optimal components for study and potential therapeutic targeting. In this study, BCA2 was analyzed in the context of its expression in breast cancer tissues and the relationship between the location of BCA2 or hHR23a expression and variables considered being of diagnostic and prognostic value. Of the patient attributes analyzed, only grade had a statistically significant correlation with nuclear BCA2 expression, such that a mid- level (2) Grade classification was correlated with high BCA2 expression in the nucleus. Along with previous studies which correlated overall increased BCA2 expression with ER-positive status, negative nodal status and increased survival over 5 years [2]; it is more likely that BCA2 is indirectly involved in progression of breast cancer to highly invasive and metastatic disease, rather than a driver of the process. Moreover, when hHR23a expression was analyzed against the same attributes, it was found that hHR23a correlated with ER-positive status, a correlation which had been found previously with BCA2 in a large scale (n = 1000) [2]. Also while not statistically significant, it is noteworthy that in a majority of samples, high hHR23a expression occurred in cases which were negative for lymph node status. This clinical variable was found to have correlated with high BCA2 in our previous studies [2]. The small number of significant correlations in this study may be due to the relatively small sample population, as greater emphasis is placed on individual anomalies and deviations. As ligases can be of both tumor suppressive and oncogenic natures, in order to evaluate E3s or their substrates in the context of cancer therapeutics and prognostics; an understanding of the mechanisms in which they are deregulated and/or stabilized is essential.

## Conclusion

**Figure 6:**
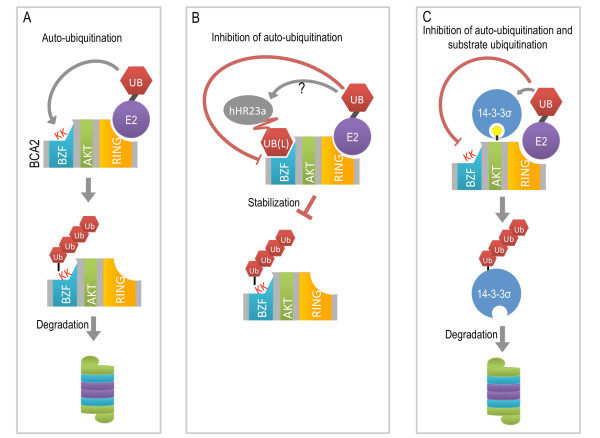
**Model for BCA2 stability**. [**A**] BCA2 in the presence of specific E2, but in the absence of binding partner or substrate is targeted to the proteasome for degradation through auto-ubiquitination in the BZF domain (as shown in Figure 2). [**B**] Depicts the potential interaction between BCA2 and hHR23a. We speculate that the ubiquitin-like (UBL) domain of hHR23a binds to the ubiquitin-binding domain (BZF) of BCA2, which not only leads to protein stabilization but also plays a role as a scaffold for ubiquitination of other substrates to aid BCA2 in its role as an E3 ligase. [**C**] 14-3-3σ binds BCA2 in the AKT phosphorylation domain which contains two 14-3-3 binding motifs, the result of binding is possible steric inhibition of the autoubiquitination of BCA2, leaving the RING domain of BCA2 available to facilitate the conjugation of ubiquitin to 14-3-3σ.

The regulation mechanisms of E3 ligases are still veritable "black boxes". Reviewed by de Bie and Ciechanover [15], there are many mechanisms by which stability, turnover, substrate affinity and ubiquitination are controlled. c-Cbl, a RING-type E3 responsible for the internalization and degradation of EGFR, has been found to be activated to a ubiquitination-capable state by phosphorylation [41]. Moreover, dynamic mono-ubiquitination or non-K48 linked multi- and branched multi-ubiquitin chains, can also either increase the stability of the E3 ligase or change its ability to bind substrate as is the case with E3 ligases RING1B and histone H2A [42,43]. But largely, the most observed method of E3 regulation, or deregulation, is proteolytic targeting through auto-ubiquitination. It has been observed in many E3 ligases that the presence of substrate or other interacting partners inhibit and attenuate the proteins self-destructive activity [44]. Interestingly, E3 ligases interact with other components of the ubiquitin proteasome system often with proteins that have antagonistic roles. One such example is ICP0, a RING-type E3 ligase which stimulates lytic infection of Herpes Simplex virus-1. Upon binding the de-ubiquitinase USP7, the ability of ICP0 to auto-ubiquitinate is counteracted, and is subsequently unable to target itself for degradation and thus stabilized [44]. In other instances, E3 ligases form heterodimers, such as BRCA1/BARD1 or LMP-1 and TRAF3. Such interactions may have functional consequences; they also result in the stabilization of the interacting E3's and prevent auto-ubiquitination, as is the case of LMP-1 [45]. From the experiments performed in this study and knowledge of how other E3 ligases respond to a similar problem with inherent protein stability, we propose the following model for the likely behavior of BCA2 (Figure 6). In the absence of a binding partner or substrate protein, BCA2 likely catalyzes its own ubiquitination, where the RING domain facilitates the addition and elongation of ubiquitin chain to the known accepting lysines in the BZF domain [7] (Figure 6A). When a target or interacting protein such as hHR23a binds, likely in the BZF domain, and results in the inhibition of autoubiquitination activity which in turn stabilizes the BCA2 E3 ligase (Figure 6B). This interaction prolongs BCA2 half-life and would then allow for the ubiquitination of a substrate, or incorporation into a complex. Due to the affinity of the BZF domain to ubiquitin, a confirmed binding protein, and also to UBA52 [7], a ubiquitin fusion protein, we propose that a substrate for BCA2 may be previously mono-ubiquitinated or containing a ubiquitin like-domain. In the case of 14-3-3σ, the interaction between phosphorylated BCA2 and 14-3-3σ, prevents self-ubiquitin conjugation by BCA2, and results in the catalysis of poly-ubiquitin chain formation and elongation on 14-3-3σ, followed by degradation (Figure 6C).

Our previous work found BCA2 expression correlates with positive estrogen receptor status, negative lymph node status with an increase in disease-free survival for regional recurrence. In this study we found that hHR23a is also correlated with ER-positive status and trends towards negative nodal status. Now we have found that high BCA2 levels, specifically in the nucleus also correlates with tumor grade, and that the ability of BCA2 to be up-regulated is likely due to presence of hHR23a or 14-3-3σ. Together these clinical associations indicate that the mechanisms of BCA2 regulation may be important to the physiology of breast carcinogenesis and growth, as well as the predictability of tumor response to treatment.

## Competing interests

The authors declare that they have no competing interests.

## Authors' contributions

SB carried out molecular studies and statistical analysis as well as drafted the manuscript. YA performed molecular and genetics studies. WY performed both IHC and IF studies. JZ scored tissue array staining. AB participated in design and conception of this study. MY aided with imaging. AKS formulated the design and concept of the study, and helped to draft the manuscript. All authors read and approved the final manuscript.

## Pre-publication history

The pre-publication history for this paper can be accessed here:

http://www.biomedcentral.com/1471-2407/12/63/prepub
